# Physiological Responses of Apple to Nitrogen Fertilization Regimes: Roles of Calcium Metabolism in Fruit Quality and Bitter Pit Development

**DOI:** 10.3390/plants15121820

**Published:** 2026-06-12

**Authors:** Yue Xing, Zhanling Zhu, Ge Tian, Minghui Du, Hui Cao, Shunfeng Ge

**Affiliations:** 1 College of Life Sciences, Zaozhuang University, Zaozhuang 277160, China; xingyue@uzz.edu.cn (Y.X.);; 2 College of Horticulture Science and Engineering, Shandong Agricultural University, Tai’an 271018, China; 3 College of Horticulture, Shanxi Agricultural University, Taiyuan 030031, China

**Keywords:** apple, N supply, Ca availability, Ca allocation, fruit quality

## Abstract

Excessive or improper nitrogen (N) fertilization can disrupt calcium (Ca) nutrition in apple trees and induce Ca-related physiological disorders, yet its effects on Ca availability and partitioning remain unclear. This study evaluated the impact of different N fertilization regimes on soil Ca availability, Ca partitioning, and Ca bioavailability in fruit tissues of 10-year-old ‘Fuji’ apple trees, using Ca fractionation analysis combined with multi-criteria decision-making (TOPSIS). High N applied as a single dose (H1) significantly reduced soil water-soluble and exchangeable Ca, while increasing Ca oxalate (CaOx) accumulation in fruit pedicels, particularly at maturity. Although total CaOx in fruit flesh decreased, its relative proportion increased, indicating enhanced Ca sequestration. In contrast, split application of moderate N (M3) maintained more stable soil Ca availability, reduced CaOx accumulation, and improved Ca allocation to fruit tissues. Integrated evaluation ranked treatments as M3 > M1 > H3 > H1. Overall, moderate and split N fertilization reduced Ca sequestration into CaOx, enhanced Ca availability, and improved Ca distribution in fruit tissues, providing a physiological basis for optimizing N management to mitigate Ca-related disorders and improve fruit quality.

## 1. Introduction

The increasing demand for high-quality horticultural products has intensified the need to understand the physiological and nutritional factors regulating fruit quality and storability. Among these, nutrient imbalance is a major constraint affecting fruit development, physiological processes, and postharvest performance [1]. Therefore, elucidating plant responses to such imbalances is essential for improving productivity and sustainability. Calcium (Ca) has a major impact on fruit quality by maintaining cell wall structure, stabilizing membranes and functioning as a key secondary messenger in stress responses [1,2]. In addition to its structural role, Ca homeostasis in plants is tightly regulated by multiple Ca-related transporters and signaling genes. Ca transport and compartmentation are mediated by channels and transport proteins such as Ca^2+^-ATPases, Ca^2+^/H^+^ exchangers (CAXs), cyclic nucleotide-gated channels (CNGCs), and glutamate receptor-like channels (GLRs) [2,3]. These genes participate in Ca uptake, long-distance transport, and vacuolar sequestration, thereby influencing Ca distribution and physiological availability in fruit tissues. Adequate Ca accumulation enhances fruit firmness and storability, whereas Ca deficiency or improper distribution leads to internal fruit disorders including bitter pit, watercore, and cortical browning [3]. Notably, these disorders are more closely associated with Ca distribution and functional forms than with total Ca content. Due to its limited phloem mobility, Ca transport relies primarily on xylem flow, making fruits particularly susceptible to Ca deficiency even under sufficient soil Ca supply. Therefore, during the peak period of calcium absorption, it is necessary to provide sufficient calcium while maintaining a balanced ratio of essential nutrients to meet the specific needs of each organ of the apple tree [4].

Nitrogen (N) is widely applied to promote vegetative growth and yield; however, excessive N fertilization is common in intensive production systems and often disrupts nutrient balance. High N supply stimulates vigorous vegetative growth, increasing competition for Ca between shoots and fruits, while also inhibiting Ca uptake and transport through alterations in root physiology and ion interactions [5,6]. As a result, excessive N has been closely linked to increased occurrence of Ca-associated disorders like bitter pit [6]. Given these complexities, optimizing N management strategies is essential for maintaining nutrient balance and improving fruit quality. Split N application, compared with single high-dose application, has been suggested as a more effective approach to synchronize N supply with plant demand, reduce excessive vegetative growth, and improve nutrient use efficiency [7,8]. However, its effects on Ca availability in the soil–plant system, as well as on Ca forms and partitioning within fruit tissues, remain poorly understood.

In fruit tissues, Ca exists in various forms, such as water-soluble Ca, pectin-bound Ca, protein-bound Ca, and calcium oxalate (CaOx) [9,10]. Among these, water-soluble Ca is the most physiologically active, whereas CaOx represents an inactive or sequestered form [11]. It has been proposed that excessive N supply may promote the conversion of Ca into CaOx, thereby reducing its functional availability [9], although this mechanism remains insufficiently validated under field conditions. Recent studies have suggested that bitter pit is not only associated with total fruit Ca concentration but also with Ca distribution, mobility, and physiological availability. Oxalate could chelate Ca and promote the formation of insoluble CaOx, which may reduce the pool of available Ca required for membrane stability and cell wall integrity [8]. In apple, CaOx accumulation in fruit-related tissues may restrict Ca transport and contribute to localized Ca deficiency, thereby increasing the risk of bitter pit development [10,11]. Moreover, long-term over-application of N fertilizers could lead to soil acidification and enhanced leaching of base cations, further limiting Ca availability and uptake [12,13]. Thus, Ca deficiency in fruit often results from impaired transport and utilization rather than soil shortage alone [5]. Optimizing N management is therefore crucial for maintaining nutrient balance and improving fruit quality [14]. Split N application has been suggested as a strategy to better match plant demand and enhance nutrient use efficiency, yet its effects on Ca availability and partitioning in fruit tissues remain unclear.

In this study, we investigated how different N fertilization regimes (single versus split application) regulate Ca availability in the soil–plant system and influence Ca distribution and its chemical forms in ‘Fuji’ apple fruit. We hypothesized that excessive single N application promotes Ca sequestration into inactive forms, whereas split N application maintains higher levels of metabolically active Ca. These findings aim to provide a physiological basis for optimizing fertilization strategies and improving fruit quality.

## 2. Results

### 2.1. Soil Ca Forms

**Figure 1:**
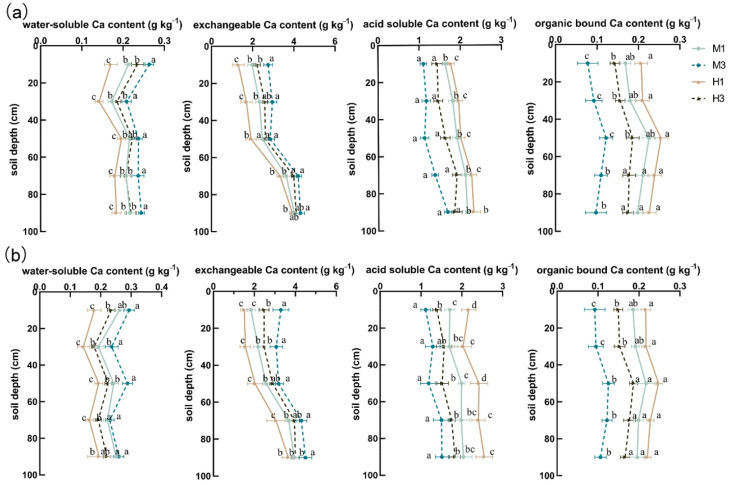
Effects of split and stabilized nitrogen supply on Ca forms in different soil layers. (**a**) Variations in water-soluble Ca, exchangeable Ca, acid-soluble Ca, and organically bound Ca in the 0–100 cm soil profile in 2023; (**b**) variations in the four Ca forms in the 0–100 cm soil profile in 2024. Different letters indicate significant differences at *p* < 0.

Different N fertilization regimes significantly affected the distribution of soil Ca forms along the 0–100 cm profile (Figure 1). Similar trends were observed in 2023 and 2024, with more pronounced differences in 2024. Water-soluble Ca (H_2_O-Ca) declined in the 0–40 cm soil layer depth and then gradually elevated and stabilized in the 40–100 cm. The H1 treatment consistently showed lower H_2_O-Ca levels than the other treatments, whereas M3 maintained the highest levels. Exchangeable Ca increased slightly in the 0–40 cm soil depth and more markedly in the 40–100 cm zone before stabilizing. In the topsoil (0–40 cm), exchangeable Ca was highest under M3, followed by H3 and M1, and lowest under H1, while no clear differences were observed in deeper layers. Acid-soluble Ca remained relatively stable in the surface layer but elevated along the vertical soil profile. Organically bound Ca increased in the 0–60 cm layer and decreased in the 60–100 cm, with the highest levels observed under M3 and the lowest under H1.

### 2.2. Ca Accumulation in Different Organs and Ca-Related Gene Expression

**Figure 2:**
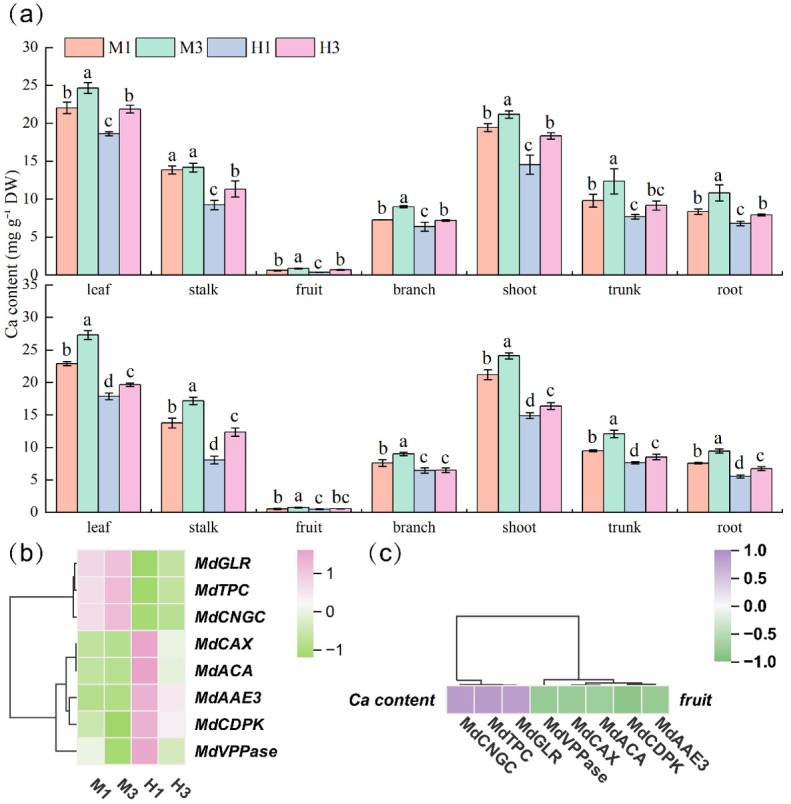
The impacts of different nitrogen fertilization applications on Ca content and Ca-related gene expression in apple fruit. (**a**) Ca content in fruit at the mature stage; (**b**) the abundance of gene expression associated with Ca in fruit flesh; (**c**) correlation between the gene expression of Ca-related genes and Ca content in fruit flesh. Different letters indicate significant differences at *p* < 0.

Ca accumulation patterns among different organs were consistent across both years (Figure 2). Ca content was highest in leaves, perennial branches, and pedicels, followed by roots and trunks, and lowest in fruit tissues. Among treatments, M3 showed the highest Ca content in leaves, pedicels, and fruit, whereas H1 exhibited the lowest values across all organs.

Investigation of the expression of Ca-associated genesin fruit flesh revealed that Ca-receptor-related genes (*MdGLR*, *MdTPC*, and *MdCNGC*) were more highly expressed under M1 and M3 treatments. In contrast, Ca-transport-related genes (*MdCAX*, *MdACA*, *MdAAE3*, *MdCDPK*, and *MdVPPase*) showed higher expression under H1. A significant negative association was identified between Ca content and the expression of Ca-transport-related genes (Figure 2c).

### 2.3. Ca Content and Forms in Young Fruit

**Figure 3:**
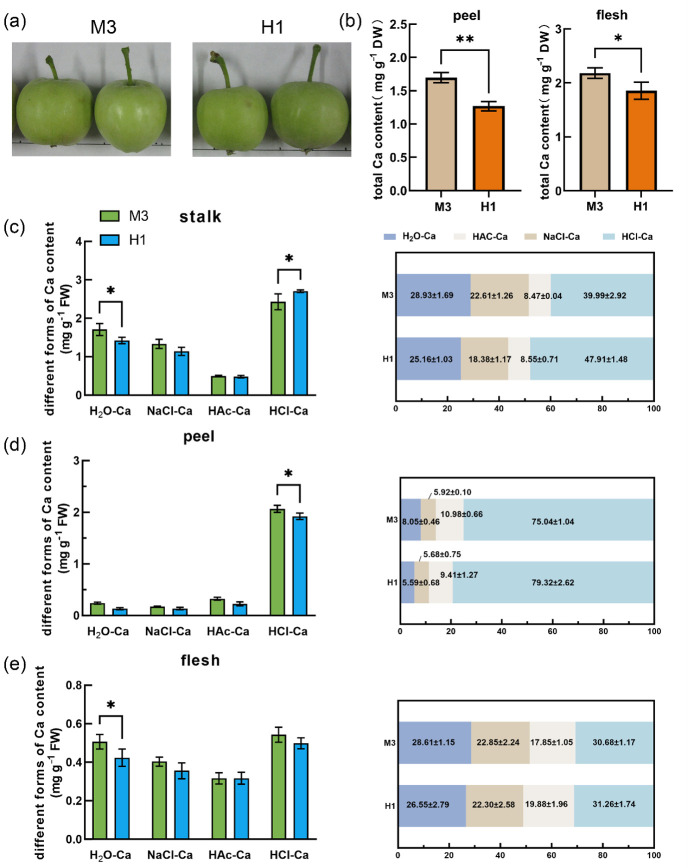
Calcium (Ca) content and forms in young apple fruit. (**a**) Fruit phenotypes under M3 and H1 treatments; (**b**) total Ca content in peel and flesh; (**c**) content and percentage of different Ca forms within the pedicel; (**d**) content and proportion of various Ca forms in the peel; (**e**) content and percentage of different Ca forms in the flesh. * indicates *p* < 0.05, and ** indicates *p* < 0.01.

In young fruit, the total Ca concentration in the peel exhibited a notably higher value under M1 than under H1 (Figure 3). In the pedicel, H1 reduced water-soluble Ca and increased CaOx accumulation compared with M3, with CaOx accounting for 47.91% of total Ca. In the peel, although CaOx content was lower under H1 than M3, its relative proportion was higher, while other Ca forms showed no significant differences. Within the fruit flesh, the concentration of water-soluble Ca was significantly lower under H1 than M3, accounting for 26.55% and 28.61%, respectively, whereas no significant differences were observed in CaOx or other Ca forms.

### 2.4. Ca Forms in Mature Fruit

**Figure 4:**
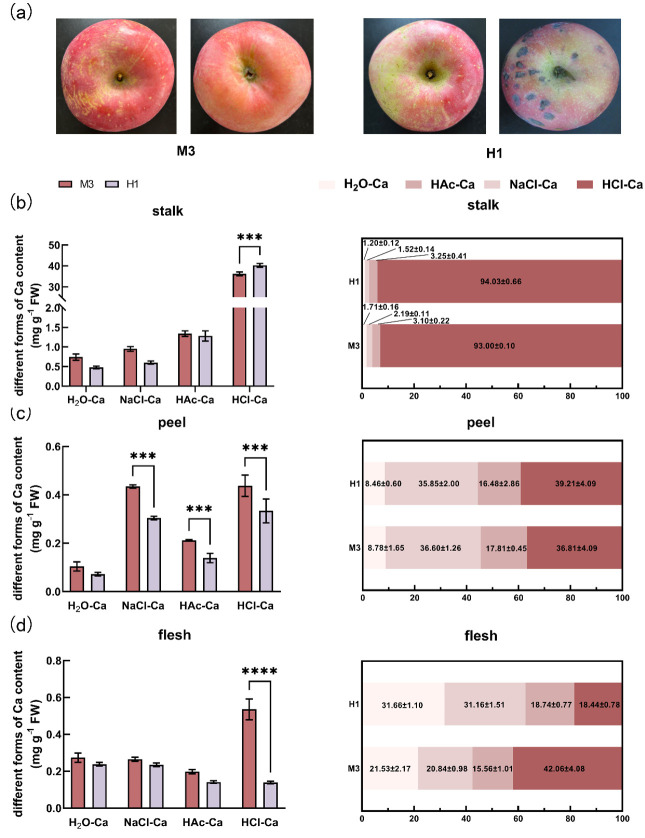
Effects of different nitrogen fertilization regimes on Ca forms in mature apple fruit. (**a**) Representative fruit phenotypes under M3 and H1 treatments; (**b**) content and percentage of different Ca forms within the pedicel; (**c**) content and percentage of different Ca forms within the peel; (**d**) content and percentage of different Ca forms within the flesh. *** indicates a *p*-value less than 0.001, and **** indicates a *p*-value less than 0.0001.

Fruit under the M3 treatment showed normal phenotypes, whereas H1 exhibited typical Ca deficiency symptoms, including bitter pit (Figure 4). In the pedicel, Ca was predominantly present as CaOx, with a significantly higher proportion under H1 (94.03%) than M3. In the peel, H1 showed lower content of pectin-bound Ca, phosphate-associated Ca, and CaOx compared with M3, although the relative proportion of CaOx was higher. In the flesh, only CaOx differed significantly among treatments, while other Ca forms remained unchanged. The proportion of CaOx under M3 reached 42.06%.

### 2.5. Incidence and Severity of Bitter Pit

**Figure 5:**
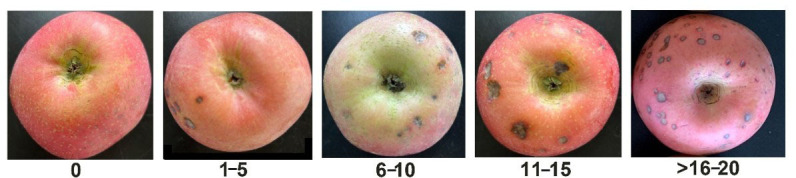
Grading bitter pit severity in apple fruit. The scoring standards for 0, 1, 2, 3, and 4 points were as follows: no lesion, 1–5, 6–10, 11–15, and 16–20 lesions, respectively.

Nitrogen fertilization significantly affected the occurrence rate as well as the severity of bitter pit (Figure 5; Table 1). Compared with H1, the M3 treatment led to a reduction in the occurrence rate of bitter pit by 57.66% and 64.46% in 2023 and 2024, respectively, and decreased severity by 63.80% and 73.10%. Across treatments, bitter pit incidence and severity ranked as M3 > M1 > H3 > H1, with H1 showing the highest values.

### 2.6. Fruit Appearance and Internal Quality

**Figure 6:**
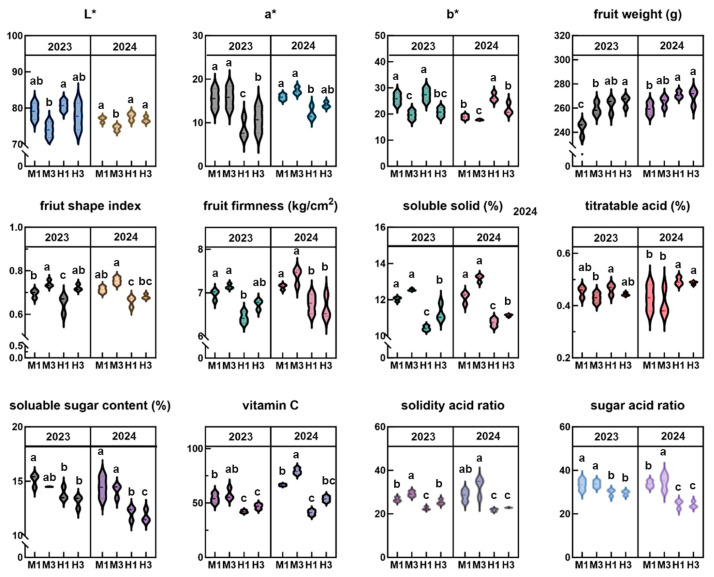
Fruit quality parameters in 2023 and 2024. L*, a*, and b* color values; single fruit weight; fruit shape index; fruit firmness; soluble solids content; titratable acidity; soluble sugar content; vitamin C content; soluble solids-to-acid ratio and sugar-to-acid ratio. Different lowercase letters indicate significant differences among treatments at *p* < 0.05 according to one-way ANOVA followed by Duncan’s multiple range test.

Fruit quality parameters were significantly influenced by N fertilization regimes (Figure 6). The M3 treatment showed higher a* values, whereas H1 exhibited higher L* and b* values. Single fruit weight was the highest under M3, followed by H3 and H1. The fruit shape index was also higher under M3. Fruit firmness, total soluble solids, and vitamin C content demonstrated a significant elevation under M3, followed by H3 and M1, and the lowest under H1. There were no significant changes in the soluble sugar levels. Titratable acidity was lower under M3, while the soluble solids-to-acid ratio and sugar-to-acid ratio were higher, increasing by 9.76%, 11.47%, and 38.10%, respectively, compared with H1.

### 2.7. Comprehensive Evaluation Using the TOPSIS Method

The TOPSIS analysis showed that treatments were ranked as M3 (0.999) > M1 (0.554) > H3 (0.426) > H1 (0.098), indicating that M3 achieved the best overall performance in fruit quality (Table 2).

## 3. Discussion

### 3.1. Effects of Split N Application on Soil Ca Forms

N fertilization regimes exert a crucial influence on the regulation of the transformation and balance of soil Ca pools in orchard systems. Soil available Ca mainly comprises water-soluble and exchangeable forms, among which water-soluble Ca represents the most readily absorbable fraction and reflects the dynamic status of the soil Ca pool due to its high mobility and transformation potential [15]. In this study, distinct vertical distribution patterns of water-soluble, exchangeable, and acid-soluble Ca were observed across the soil profile, with decreasing contents in the 0–40 cm layer followed by gradual increases in the 40–100 cm zone. Previous studies have indicated that N is susceptible to losses via runoff and deep leaching, whereas split application can mitigate fluctuations in soil N concentration and ensure a more stable nutrient supply during later stages of fruit development [16,17]. In contrast, single high-dose N application often leads to nutrient imbalance and accelerates soil acidification and secondary salinization [18]. These processes may enhance the dissolution and leaching of Ca and other secondary nutrients or promote their conversion into less available forms, thereby impairing Ca uptake and disrupting physiological processes in fruit trees. Consistent with these findings, the present study showed that single high N application accelerated the depletion of water-soluble and exchangeable Ca while promoting their transformation into acid-soluble Ca. In contrast, split N application enhanced the conversion of acid-soluble Ca into more available forms, thereby increasing the levels of water-soluble and exchangeable Ca, particularly in the topsoil (0–40 cm), where management practices exert the greatest influence. This suggests that split N application improves soil Ca availability by facilitating the transformation of less available Ca pools into more bioavailable forms.

### 3.2. Effects of Split N Application on Bitter Pit and Fruit Quality

Bitter pit symptoms were observed to develop in September and were closely associated with N application during the fruit expansion stage (July–September) [19]. Single N application, typically conducted at early growth stages, can rapidly increase tree N status and promote vigorous vegetative growth. In orchards with vigorous vegetative growth, split N application should also be integrated with pruning, canopy management, and crop load regulation to promote Ca transport to fruit and reduce the risk of bitter pit; however, it may also intensify the antagonistic interaction between N and Ca, thereby reducing Ca accumulation in fruit [20,21]. Excessive N fertilization has been widely reported to elevate the occurrence rate of physiological disorders, including bitter pit and fruit cracking, primarily due to reduced Ca uptake and impaired Ca transport to fruit, particularly during fruit enlargement [22,23]. Previous studies have shown that apple pedicels contain relatively high levels of oxalate, which can combine with Ca to form CaOx, thereby restricting Ca transport into fruit; this effect becomes more pronounced as fruit develops. CaOx accumulation may primarily affect Ca partitioning within fruit tissues, resulting in uneven Ca distribution and an imbalance in Ca metabolism [24]. This altered Ca allocation and reduced the availability of physiologically active Ca in specific fruit regions, leading to localized Ca deficiency and ultimately contributing to bitter pit development [22,25]. Differences in Ca speciation between healthy and bitter-pit-affected fruits are largely associated with CaOx accumulation [25]. In the present study, significant differences in CaOx content in the pedicel were observed between affected and healthy fruit at the young fruit stage, whereas no significant differences were detected in the flesh [9]. At maturity, CaOx content in the pedicel under single N application was greater than that detected in normal fruit (*p* < 0.001), and differences in flesh CaOx became more pronounced.

These results suggest that single N application promotes CaOx precipitation in the pedicel, thereby increasing resistance to Ca transport into the fruit [2]. In contrast, moderate split N application may facilitate the conversion of CaOx into more soluble Ca forms, thereby enhancing Ca availability and increasing Ca accumulation in the flesh. At the molecular level, split N application was associated with higher expression of Ca^2+^ channel-related genes (*MdGLR*, *MdTPC*, and *MdCNGC*), which may promote Ca uptake and transport [26,27]. Conversely, single N application upregulated genes involved in vacuolar Ca transport and sequestration (*MdCAX*, *MdACA*, *MdAAE3*, *MdCDPK*, and *MdVPPase*), indicating enhanced sequestration of cytosolic Ca into vacuoles and subsequent CaOx formation [19]. This process likely reduces cytosolic Ca availability and contributes to Ca deficiency symptoms. Furthermore, CaOx precipitation in the pedicel may act as a physical and physiological barrier to Ca translocation into the fruit flesh, thereby exacerbating Ca deficiency. Consistently, comprehensive fruit quality analysis revealed that moderate split N application brought about a significant improvement in both external and internal fruit quality, likely through enhancing Ca availability and optimizing Ca distribution within the soil–plant system.

## 4. Materials and Methods

### 4.1. Experimental Site and Design

The experiment was conducted in 2023 and 2024 in an orchard located in Guanzhuang Village, Laishan Town, Yantai, Shandong Province, China. Ten-year-old ‘Fuji’ apple trees (*Malus domestica* Borkh.) grafted onto M.26 with *Malus domestica* Rehd. as the interstock were used. In 2012, the trees were planted with a 1.5 m × 4 m spacing.

The soil was classified as loam, with the following properties: organic carbon, 8.02 g kg^−1^; nitrate-N, 27.13 mg kg^−1^; ammonium-N, 19.82 mg kg^−1^; available P, 31.38 mg kg^−1^; and available K, 231.32 mg kg^−1^. Soil bulk density in the 0–100 cm profile ranged from 1.14 to 1.46 g cm^−3^. Twenty-four trees with uniform growth and no visible pests or diseases were selected and arranged in a 2 × 2 factorial design (N rate × application method), with six replicates per treatment (one tree per replicate). The treatments were as follows: M1 (moderate N, single application): 150 g Ca(NO_3_)_2_ per tree applied once on March 20; M3 (moderate N, split application): 50 g Ca(NO_3_)_2_ per tree applied on March 20, July 10, and August 25; H1 (high N, single application): 300 g Ca(NO_3_)_2_ per tree applied once on March 20; H3 (high N, split application): 100 g Ca(NO_3_)_2_ per tree applied on March 20, July 10, and August 25.

Fertilizer was applied into a circular trench (20 cm in depth and width) at a distance of 40 cm from the trunk. Fruit samples were collected at the first fruit expansion stage (June 20) and at maturity (185 days after full bloom). Six fruits per tree were randomly sampled from the middle outer canopy in four directions (n = 36 per treatment). Peel and flesh were separated, then promptly frozen in liquid nitrogen, and subsequently stored at −80 °C. The soil samples were collected outside the fertilization zone within the canopy projection area. Six sampling points per tree were taken at 20 cm intervals down to a depth of 100 cm, and samples from each layer were composited for analysis.

### 4.2. Determination of Ca Forms in Plant Tissues

Ca forms in plant tissues were determined using a sequential extraction method with slight modifications [28]. Briefly, fresh plant samples (1.0 g) were homogenized and sequentially extracted with deionized water, 1 mol L^−1^ NaCl, 2% acetic acid, and 5% HCl to separate Ca into four operationally defined fractions. The deionized water extract represented water-soluble Ca (H_2_O-Ca), which mainly includes free Ca^2+^ and readily soluble Ca salts. The NaCl extract represented salt-soluble or exchangeable Ca (NaCl-Ca), primarily associated with pectates and weakly bound Ca complexes. The 2% acetic acid extract represented acetic-acid-soluble Ca (HAC-Ca), including Ca phosphates and other moderately soluble Ca compounds. The 5% HCl extract represented hydrochloric-acid-soluble Ca (HCl-Ca), mainly corresponding to insoluble Ca oxalate and other strongly bound Ca forms.

After each extraction step, the homogenate was centrifuged or filtered, and the supernatant was collected for Ca determination. To minimize chemical interference during measurement, 5% lanthanum chloride was added to each extract prior to analysis. Calcium concentrations in all fractions were then quantified using an atomic absorption spectrophotometer (ZEEnit700P, Analytic Jena, Germany).

### 4.3. Ca-Related Gene qRT-PCR Assays

The reaction mixture (20 μL) contained the following: 10 μL of SYBR Green Supermix, 7 μL of ultra-pure water, 1 μL of cDNA template, and 1 μL of each forward and reverse primer. The relative abundance of Ca-related genes was determined via the 2^−∆∆^Ct method, with *MdActin* as the internal reference. All primer sequences are detailed in Table 3.

### 4.4. Determination of Soil Ca Forms

A sequential extraction technique was employed to determine the different forms of soil Ca. Water-soluble Ca was extracted with distilled water (shaken for 1 h), exchangeable Ca with ammonium acetate (shaken for 2 h), and organically bound Ca with 3 mol L^−1^ HCl at 80 °C for 2 h. Residual Ca was determined after HF–HClO_4_ digestion of the remaining residue. All Ca fractions were quantified by atomic absorption spectrophotometry [10].

### 4.5. Assessment of Bitter Pit Severity

Bitter pit severity was evaluated using 30 fruits per treatment. Fruits were classified into five grades based on lesion number (0, 1–5, 6–10, 11–15, and >16 lesions). The bitter pit severity index (BPSI) was conducted according to the following formula:

BPSI = Σ (severity grade × number of fruits at that grade)/(total number of fruits × maximum grade)

### 4.6. Fruit Quality Measurements

Fruit color parameters (L*, a*, b*) were recorded at the equatorial region using a colorimeter (NR145, Shenzhen, China), and the hue angle (h°) was calculated as arctan (b*/a*). Fruit size was assessed using a digital caliper, and the shape index was determined as the ratio of longitudinal diameter to transverse diameter.

Fruit firmness was assessed through the utilization of a texture analyzer (TA-HD Plus, Godalming, UK), which was equipped with a 10 mm probe. Total soluble solids were determined with a refractometer (PAL-1, Tokyo, Japan). The determination of titratable acidity was carried out through NaOH titration. Soluble sugar quantification was achieved using the anthrone method, and the assessment of vitamin C content was performed via 2,6-dichlorophenol indophenol titration [29,30].

### 4.7. Statistical Analysis

The statistical analyses were executed utilizing SPSS 21.0 software (IBM, Armonk, NY, USA). One-way analysis of variance (ANOVA) was employed to evaluate the differences among treatments. Graphs were prepared with Origin 8.0 and GraphPad Prism 9 software. The data are presented as mean values ± standard deviation (SD), calculated from three independent biological replicates.

## 5. Conclusions

Nitrogen fertilization strategy strongly influences Ca dynamics and fruit quality in ‘Red Fuji’ apple. Single, high-dose N application reduced soil Ca availability and promoted Ca sequestration in the form of CaOx, resulting in decreased Ca availability in fruit and increased incidence of bitter pit. In contrast, moderate split N application maintained soil Ca availability, reduced CaOx accumulation, and improved Ca allocation to fruit tissues. Consequently, split N application enhanced fruit quality and mitigated Ca-deficiency-related disorders.

Therefore, in practical ‘Red Fuji’ apple production, excessive single N application should be avoided, and moderate split N application should be adopted according to local soil Ca status and precipitation conditions. Optimal effectiveness is obtained by applying it three times: before flowering, after petal fall, and during the fruit expansion stage. In regions with low soil Ca availability or frequent rainfall, N management should be combined with soil testing, Ca supplementation, and appropriate irrigation regulation. Considering factors such as cultivar and soil conditions, the findings of this study have certain limitations. More extensive trials are needed to determine whether the results are applicable to other regions.

## Figures and Tables

**Table 1:** Bitter pit incidence and severity.

Treatments	2023		2024	
BB	Occurrence Rate (%)	Severity Index (0–100)×	Occurrence Rate (%)	Severity Index (0–100)×
M1	15.5 ± 1.32 b	7.9	12.5 ± 0.5 c	6.9
M3	9.4 ± 0.86 c	6.2	8.6 ± 0.2 d	4.6
H1	22.2 ± 2.11 a	16.7	24.2 ± 1.65 a	17.7
H3	21.0 ± 1.16 a	14.3	18.4 ± 0.23 b	13.8

Different letters indicate significant differences at *p* < 0, equal letters indicate absence of statistical significance differences.

**Table 2:** The TOPSIS-calculated results.

Treatments	Positive-Ideal Solution D+	Negative-Ideal Solution D−	Relative Closeness C	Sequencing Results
M1	1.29	1.605	0.554	2
M3	0	2.754	0.999	1
H1	2.687	0.292	0.098	4
H3	1.783	1.325	0.426	3

**Table 3:** Primer sequences for qRT-PCR.

Gene Name	Forward Primer Sequence (5′-3′)	Reverse Primer Sequence (5′-3′)
*MdGLR2*	CAGAGACTGGTTCTGCACGT	TCGATGTCCCTCCTGTGAGT
*MdTPC1*	TCCTTACCAGAAAGCTGCCG	AAGCATTCCAGCCACGATGA
*MdCNGC2*	CGGGGTGCCAATAAGCATTC	CGTTCCTTGTTCTTGATTTGTGC
*MdCAX1*	TTACACCGGTCCAACAGTGG	TGTGTTGGGCTAGGGCAAAT
*MdACA11*	CCAGCTTCATCACCAAGGCT	AGGAAGCGCTTCATATCCGT
*MdAAE3.1*	GACATGGTCGCATACAACGC	GGCAACCTCTGGATGTGACA
*MdVPPase15*	AGAGAACGTTGCGAGCATGA	CAGAAGTATCGAGGGTCGGC
*MdCDPK3*	GGCCTTCAGACCGCTTAACT	TTGCCCTTTTGCATCATGGC
*MdACTIN*	TGACCGAATGAGCAAGGAAATTACT	TACTCAGCTTTGGCAATCCACATC

## Data Availability

The original contributions presented in this study are included in the article. Further inquiries can be directed to the corresponding authors.
